# Neuronal and synaptic adaptations underlying the benefits of deep brain stimulation for Parkinson's disease

**DOI:** 10.1186/s40035-023-00390-w

**Published:** 2023-11-30

**Authors:** Wenying Xu, Jie Wang, Xin-Ni Li, Jingxue Liang, Lu Song, Yi Wu, Zhenguo Liu, Bomin Sun, Wei-Guang Li

**Affiliations:** 1 Department of Rehabilitation Medicine, Huashan Hospital, Institute for Translational Brain Research, State Key Laboratory of Medical Neurobiology and Ministry of Education Frontiers Center for Brain Science, Fudan University, Shanghai, 200032 China; 2 Department of Neurosurgery, Center for Functional Neurosurgery, Ruijin Hospital, Shanghai Jiao Tong University School of Medicine, Shanghai, 200025 China; 3 Department of Neurology, Xinhua Hospital Affiliated to Shanghai Jiao Tong University School of Medicine, Shanghai, 200092 China; 4 Ministry of Education-Shanghai Key Laboratory for Children’s Environmental Health, Xinhua Hospital Affiliated to Shanghai Jiao Tong University School of Medicine, Shanghai, 200092 China

**Keywords:** Parkinson’s disease, Deep brain stimulation, Optogenetics, Opto-DBS, Synaptic adaptation, Orthodromic effects, Antidromic effects, Long-lasting therapeutic effects

## Abstract

Deep brain stimulation (DBS) is a well-established and effective treatment for patients with advanced Parkinson's disease (PD), yet its underlying mechanisms remain enigmatic. Optogenetics, primarily conducted in animal models, provides a unique approach that allows cell type- and projection-specific modulation that mirrors the frequency-dependent stimulus effects of DBS. Opto-DBS research in animal models plays a pivotal role in unraveling the neuronal and synaptic adaptations that contribute to the efficacy of DBS in PD treatment. DBS-induced neuronal responses rely on a complex interplay between the distributions of presynaptic inputs, frequency-dependent synaptic depression, and the intrinsic excitability of postsynaptic neurons. This orchestration leads to conversion of firing patterns, enabling both antidromic and orthodromic modulation of neural circuits. Understanding these mechanisms is vital for decoding position- and programming-dependent effects of DBS. Furthermore, patterned stimulation is emerging as a promising strategy yielding long-lasting therapeutic benefits. Research on the neuronal and synaptic adaptations to DBS may pave the way for the development of more enduring and precise modulation patterns. Advanced technologies, such as adaptive DBS or directional electrodes, can also be integrated for circuit-specific neuromodulation. These insights hold the potential to greatly improve the effectiveness of DBS and advance PD treatment to new levels.

## Background

Deep brain stimulation (DBS) is a promising treatment option for individuals suffering from advanced Parkinson’s disease (PD), especially those who initially respond well to dopamine replacement therapy but develop complications such as dyskinesia and "on–off" fluctuations over time [1, 2]. In the context of PD, DBS is thought to exert its effects on specific nuclei of the basal ganglia [3, 4]. The subthalamic nucleus (STN) and the globus pallidus internus (GPi) are the most common targets for DBS in PD. DBS at these two sites has demonstrated efficacy in the treatment of cardinal motor symptoms in the "off"-medicated state of PD [5–8], with an acceptable side effect profile, including weight gain, dysarthria, and mood changes [9]. In specific cases, the ventral intermediate thalamic nucleus (Vim) and the pedunculopontine nucleus (PPN) have been targeted to address tremor-dominant PD [10, 11] and gait and postural instability [12, 13]. Despite the proven efficacy of DBS in PD by numerous clinical trials and the recommendation of target selection based on clinical features [14–18], the common mechanisms underlying the effects remain elusive [19–26].

**Fig. 1:**
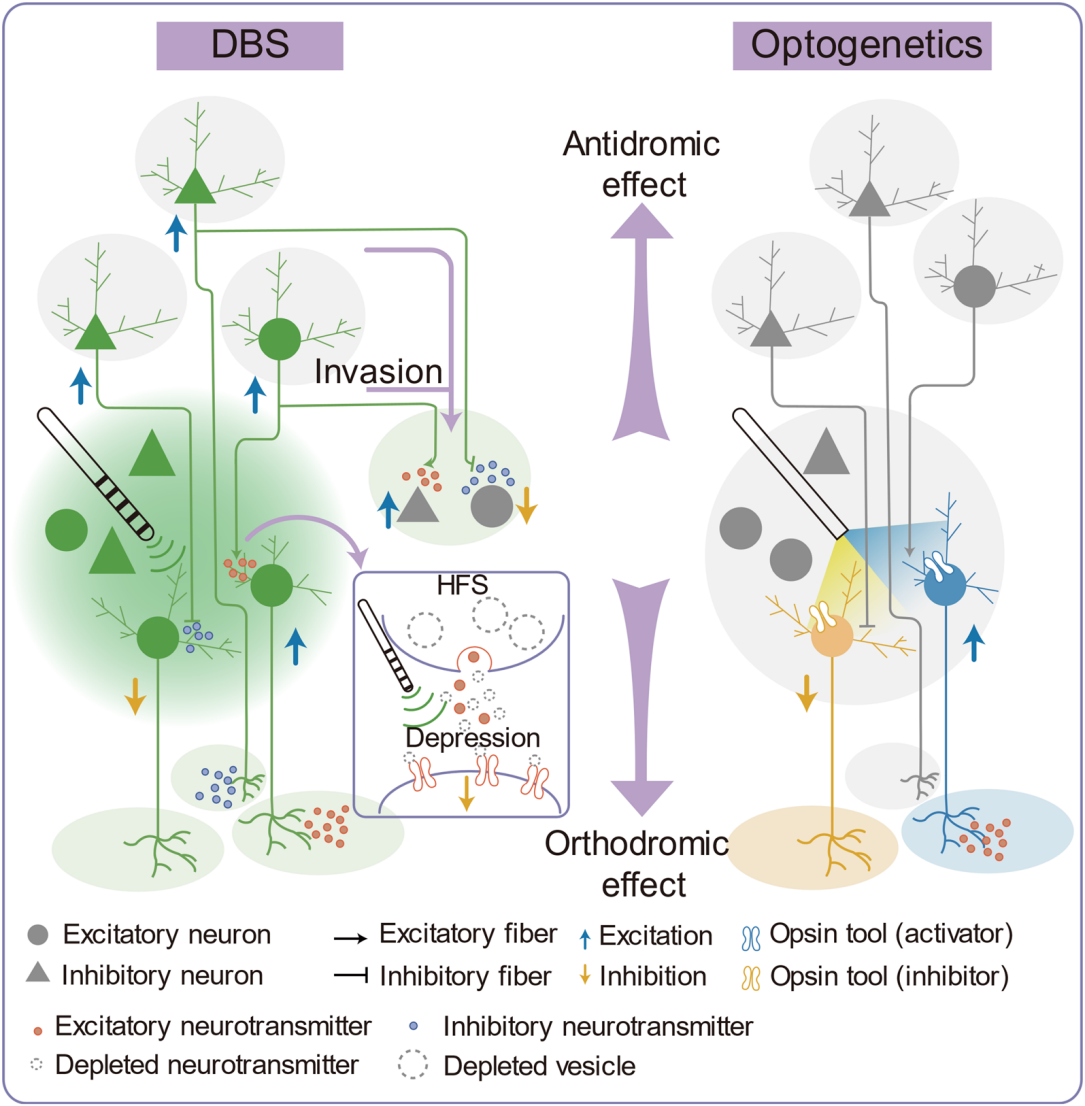
Differentiating mechanisms underlying the neuromodulation effects of DBS and optogenetics. Left: A hypothesis posits that single pulses of electrical stimuli activate all converging presynaptic inputs to stimulate target neurons. Responses at the target location are determined by the distribution of excitatory/inhibitory (E/I) afferent inputs [48, 49]. Repetitive high-frequency stimulation (HFS) can lead to neuronal suppression due to short-term synaptic depression [48, 50]. This results from rapid decreases in synaptic strength after brief bursts of activity, depleting presynaptic neurotransmitters [53]. Local action potentials (APs) evoked by the stimulus can propagate orthodromically to facilitate neurotransmitter release at the distal end of the soma and antidromically to activate upstream neurons [88]. Occasionally, the AP reaches the base of the axonal arbor first and then bifurcates at various branch points, eventually invading the entire axonal arbor and reaching all terminal points (Invasion). This leads to neurotransmitter release at terminal locations beyond the stimulation site [81, 87]. Right: In contrast, optogenetics (right) relies on genetically-encoded proteins that change conformation in response to a light stimulation, regulating cell activity [34]. Opsin tools expressed on membranes of specific neurons enable selective activation or inhibition of those neurons with light, leaving other non-opsin-expressing cells unaffected by the illumination [28]

DBS stands as a neurosurgical intervention with the potential to leverage neural adaptations for therapeutic purposes [27]. Essentially, the therapeutic benefits and possible side effects of DBS are intricately linked to the response elicited by the stimulation of cell bodies, nerve terminals, and traversing axons (Fig. 1). This stimulation sets in motion a complex interplay of functional circuits, both during and after neuromodulation [28]. Notably, these perturbations of neuromodulation can be counteracted by homeostatic plasticity mechanisms, which aim to stabilize neuronal and circuit activity [29]. This adds a layer of complexity to the overall neuronal and synaptic adaptations brought about by DBS. A comprehensive understanding of the adaptations associated with PD-DBS is pivotal for enhancing therapeutic efficacy and laying the groundwork for future applications [30] and even for achieving long-term therapeutic benefits [31–33].

Currently, optogenetics is increasingly being used in animal models to investigate the underlying mechanisms of DBS [28, 34]. Optogenetic approaches involve the introduction of light-sensitive channel proteins into target neural populations, which enables the mapping and control of neural circuits using precise light frequencies in animal models [34]. Unlike electrical stimulation, optogenetic stimulation can specifically target certain cell types, afferent and efferent projections without affecting other tissues within the stimulated area, even passing fibers (Fig. 1) [28]. In this review, we will present an overview of insights gained from opto-DBS studies regarding neural, synaptic, and circuitry aspects that explain the therapeutic benefits of DBS in PD. Our primary aim is to establish a conceptual framework for understanding the mechanisms underpinning DBS therapy.

### Local neuronal adaptations to DBS

In PD, depletion of dopamine is thought to disrupt the balance of activity between the direct and indirect pathways within the striatum [35], leading to increased activity in the indirect pathway, resulting in reduced firing in the globus pallidus externus (GPe). This, in turn, elevates activity in the STN and ultimately increases thalamic inhibition via the GPi. Simultaneously, reduced firing in the direct pathway disinhibits GPi neurons, allowing them to suppress the thalamic and cortical activity. This abnormal firing pattern within the common PD targets, STN and GPi, results in excessive inhibition of the thalamus and cortex, giving rise to symptoms like bradykinesia, rigidity, and tremor. Furthermore, evidence suggests that increased activity in the cerebellothalamocortical circuit contributes to the resting tremor observed in PD [36, 37]. Consequently, DBS targeting the Vim is effective in alleviating tremors, likely through inhibiting thalamic neurons [36–39]. While it is indisputable that DBS electrode stimulation modulates the excitability of neural tissue surrounding the electrode, it is imperative to acknowledge the multifaceted and intricate local neuronal responses at the stimulation target site.

#### Complex effects of DBS on local neuronal activity

The question of whether DBS elicits neural excitation or inhibition during treatment remains a topic of discussion. Initial experimentation supported the "inhibition hypothesis", indicating that high-frequency electrical subthalamic and pallidal stimulation enhances PD motor symptoms by obstructing overactive ganglia output and aligns with the classical rate model [40, 41]. Nonetheless, subsequent studies have uncovered a more complicated scenario (Table 1). Some studies indicated neuronal inhibition around the electrode during high-frequency stimulation (HFS) [42–44], echoing the "inhibition hypothesis", while others reported increased neural activity [45]. For instance, the responses of GPi neurons to HFS-DBS differ, with some displaying facilitation, suppression, or no change in firing rate [46, 47]. In the study by Luo et al., high-frequency microstimulation of the human GPi resulted in a prolonged after-facilitation in over one-third of neurons. In addition, the neurons exhibited two types of facilitation: continuous facilitation and discontinuous facilitation. The type of facilitation likely depended on the HFS charge density [46].

**Table 1:** Effects of DBS on neuronal activity around the electrodes

Species	Stimulation target	Stimulus parameter	Neuronal activity	Citations
PD patients (in vivo)	STN	HFS (150 μs, 20, 50 and 100 µA, 100 Hz for 10 s; 50, 150 and 250 µs, 100 μA, 100 Hz, for 5 s)	Reduced neuronal firing during HFS and prolonged post-stimulus silent periods	[71]
PD patients (in vivo)	STN	0.3 ms biphasic pulse width, 100 mA, 1–100 Hz, for 5–10 s	Decreased firing rate as the stimulation frequency was increased	[44]
PD rats (in vivo) and normal mice (ex vivo)	STN	Negative constant current injection	Decreased burst discharges	[62]
PD rats (in vivo)	STN	Optogenetic DBS using Chronos (130 pps)	Increased, decreased, and had no effects on firing rate in 53%, 32%, and 5% of neurons, respectively; eliminated oscillatory activity	[173]
PD and normal rats (in vivo)	STN	HFS (60 μs, 10–1000 μA, 130 Hz, for 5 s)	Decreased activity of all cells recorded	[42]
PD and normal rats (in vivo)	STN	HFS (60 μs, 40 μA, 130 Hz, for 10 s)	Inhibited activity of the majority of neurons	[72]
PD mice (in vivo)	STN	HFS (60 μs, 200 μA, 60 and 100 Hz)	Consistently increased activity	[45]
PD rats (in vivo)	STN	HFS (80 μs, 70 μA, 120 Hz, for 5 min)	Regularized neuronal firing patterns of PD rats, when DBS ceased	[74]
PD rats and normal rats (ex vivo)	STN	HFS (100 pulses, 100 Hz)	Depressed the amplitude of evoked EPSCs in PD, but had no effect in normal mice	[54]
PD rats and normal rats (ex vivo)	STN	HFS (60 μs, 400 μA, 130 Hz)	Decreased firing rate in both PD and normal rats; the majority of cells presented irregular or bursting pattern in PD, but regular pattern in normal rats	[72]
Normal rats (ex vivo)	STN	HFS (100 μs, 100–250 Hz, for 1 min)	Blocked ongoing neuron activity	[60]
Normal mice (ex vivo)	STN	Electrical stimuli (Unknown)	Excited 79% of α4β2^+^ neurons and inhibited 58% of α7^+^ neurons	[52]
PD patients (in vivo)	GPi	Microstimulation (0.15 ms, < 10 mA, 5 Hz)	Inhibited spontaneous activity	[43]
PD patients (in vivo)	GPi	HFS (0.1 ms,1-8 V, 88–180 Hz, for 1 min)	Decreased the mean firing rate	[77]
PD patients (in vivo) and normal rats (ex vivo)	GPi	HFS (200 μs, 10 μA and 100 μA, 333 Hz, for 10 s)	Patients: after-facilitation in 37.6% of neurons, after-suppression in 40.0% of neurons, and no change in 22.4% of neurons; decreased bursting in neurons displaying after-facilitation; Rats: after-facilitation in majority of neurons	[46]
PD rhesus monkeys (in vivo)	GPi	HFS (90 μs, 350 μA, 120 Hz, for 20 s or 120 s)	Decreased firing rate	[40]
PD macaques (in vivo)	GPi	HFS (≥ 200 μA, 150 Hz, for 30 s)	Decreased the mean firing rates; no change in burst firing; reduced prevalence of synchronized low-frequency oscillations	[47]

*EPSCs* Excitatory post synaptic currents, *GPi* Globus pallidus internus, *HFS* High-frequency stimulation, *pps* Pulses per second, *STN* Subthalamic nucleus

The variations of local neural response to HFS-DBS at the same target can be explained by a biophysically realistic computational framework [48]. This framework proposes that single pulses of electrical stimulation activate all converging presynaptic inputs to stimulate target neurons simultaneously, and the resulting responses are determined by the relative distribution of excitatory and inhibitory (E/I) afferent inputs [48, 49]. The different E/I ratios of synaptic inputs in various nuclei could account for the variable responses to DBS [50]. This framework explains that thalamic structures which mainly receive excitatory inputs show excitatory neural responses, while basal ganglia structures that mainly receive inhibitory inputs exhibit inhibitory responses [48, 51]. A similar pattern has been found by Xiao et al. [52] suggesting opposite effects on neuron subtypes following unique inputs within the targeted brain region. The STN consists of microcircuits regulated by the expression of different subtypes of nicotinic acetylcholine receptors. Local electrical stimulation mainly excites α4β2^+^ neurons and significantly inhibits 58% of α7^+^ neurons, potentially due to α4β2^+^ neurons receiving more glutamatergic inputs and α7^+^ neurons receiving more GABAergic inputs within the STN [52]. Therefore, mixed subpopulations of neurons with diverse inputs result in inconsistent, and sometimes conflicting responses to DBS at the same location.

Furthermore, computational studies suggest that repetitive HFS results in decreased site-specificity and neuronal suppression mediated by short-term synaptic depression [48, 50]. This phenomenon consists of a rapid decline in synaptic strength after brief bursts of activity, followed by a return to initial strength after a short rest period [53]. In vitro electrophysiology experiments demonstrated that HFS with 100 pulses delivered at 100 Hz significantly decreased the amplitude of evoked excitatory post-synaptic currents (eEPSCs) of STN neurons in dopamine-depleted slices. On the contrary, low-frequency stimulation (LFS) with 10 pulses per second at 40 Hz did not exhibit the same effect [54]. This short-term synaptic plasticity is a result of the depletion of readily released neurotransmitter vesicle pools when delivering rapid successive stimuli. Reduced presynaptic Ca^2+^ conductivity or inactivation of neurotransmitter release sites causes a reversible decline in synaptic efficacy due to the delayed recovery of vesicle fusion events [55–59].

In summary, at stimulation frequencies below the synaptic depression threshold, the local neuronal responses to DBS depend on the relative distributions of excitatory and inhibitory afferent inputs (site-specific effects), while at stimulation frequencies above the synaptic depression threshold, the local neuronal responses to DBS are progressively reduced, due to the synaptic depression effect (frequency-dependent effects).

#### Conversion of neuronal firing patterns by DBS

**Fig. 2:**
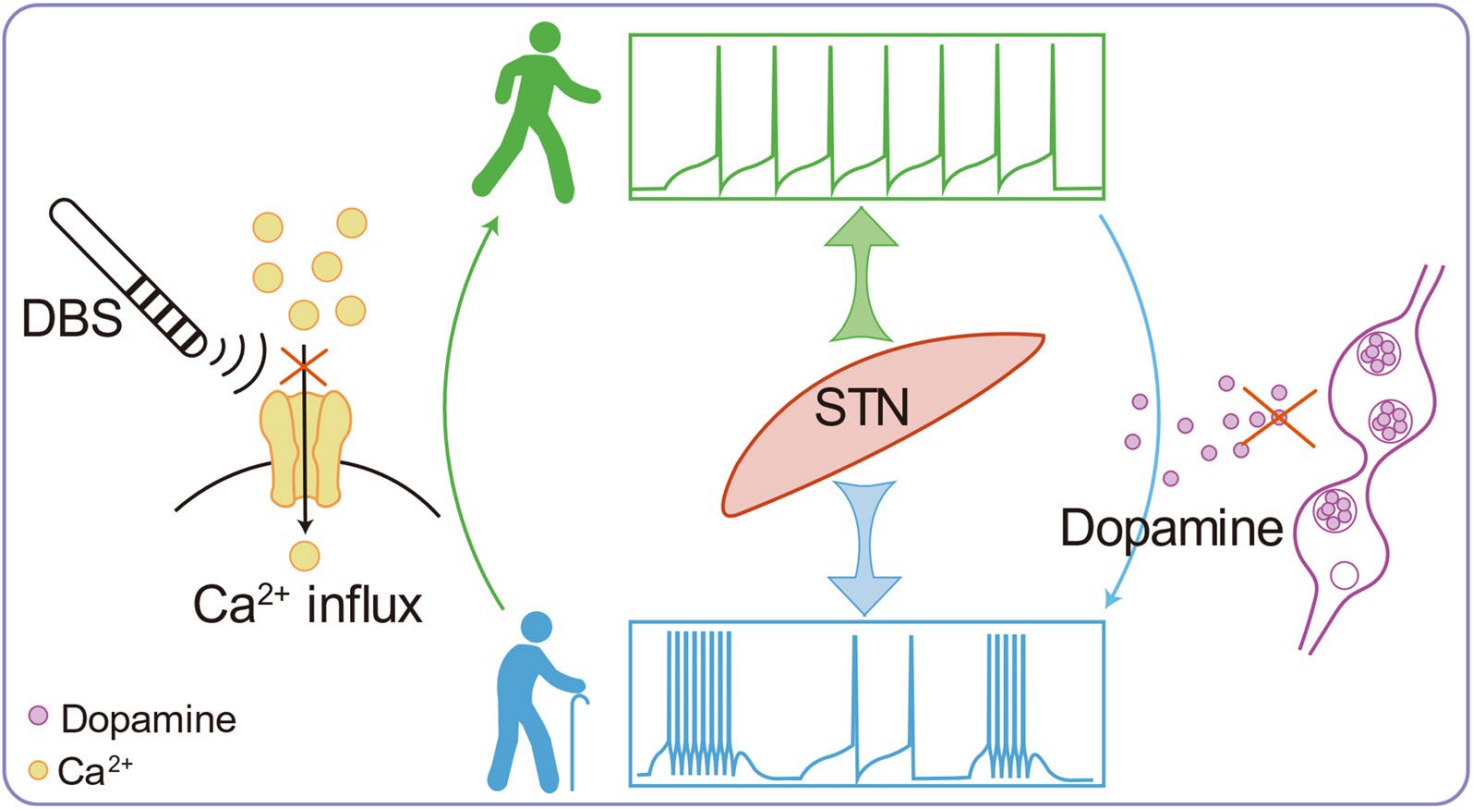
Schematic illustration of DBS suppressing abnormal burst firing in the STN. Under normal conditions, STN neurons are capable of transition between single-spike firing and burst firing by activating distinct sets of ion channels based on the membrane potential state [63]. However, in PD, dopamine deficiency results in relative membrane hyperpolarization, facilitating burst firing in the STN [64, 65]. This abnormal burst firing pattern is closely associated with the manifestation of parkinsonian symptoms [66–68]. HFS-DBS induces a transient depolarization of the neuronal membrane. Subsequently, it effectively blocks voltage-gated currents, with a notable impact on T- and L-type Ca^2+^ currents as well as Ca^2+^-activated inward currents. This suppression of abnormal burst firing in the STN contributes to the amelioration of PD symptoms [60, 73]

In addition to the intermingling of neurons exhibiting diverse responses to HFS, the modulation of neuronal firing patterns linked to intrinsic excitability further complicates the DBS effects [60–62]. For example, in STN, subthalamic neurons possess the capability to transit between single-spike firing and burst firing modes under normal conditions, a transition governed by the activation of distinct sets of ion channels contingent on the membrane's potential state [63]. However, in PD models, dopamine deficiency leads to relative membrane hyperpolarization, promoting STN burst firing. This stands in contrast to dopamine's role in depolarizing STN neuronal membranes [64, 65]. The abnormally heightened STN burst firing is intricately linked to parkinsonian symptoms [66–68] and serves both as the electrophysiological hallmark of PD and a primary target for therapeutic intervention via DBS [69]. Bursting activity in the STN has been observed to precede the pathological local field potential (LFP) oscillation in most cases, suggesting its pivotal role in generating aggregate-level LFP oscillations [70]. Therefore, amelioration of excessive STN burst firing is emerging as a fundamental mechanism underlying the clinical benefits of DBS (Fig. 2), a premise substantiated by research in PD patients, demonstrating that STN-DBS mitigates excessive burst firing and ameliorates PD symptoms [71].

Similarly, experiments with HFS in PD animals have shown that most STN neurons are inhibited and burst firing is reduced [72] (Fig. 2). Electrophysiological recordings have also shown that HFS can suppress STN burst firing through transient neuronal membrane depolarization and subsequent inhibition of voltage-gated currents, particularly T- and L-type Ca^2+^ currents and Ca^2+^-activated inward currents [60, 73]. Notably, STN burst firing can be bidirectionally regulated by altering the neuronal membrane potential using different electrical stimuli—depolarizing current (with or without pulses) decreases burst firing, whereas application of reverse-polarizing hyperpolarizing currents increases it [61, 62]. In addition, the hyperpolarization-activated cyclic nucleotide-gated channel 2 (HCN2) channels coupled to histamine H2 receptors, the GluN2A subunit-containing *N*-methyl-*D*-aspartate ionotropic receptors (NMDARs), and the ether-à-go-go-related gene (ERG) K^+^ channels have been identified as factors that can regularize neuronal firing patterns [74–76].

Firing pattern conversions have also been observed in the GPe and GPi. Studies in unanesthetized patients have shown that DBS in the GPi does not uniformly silence local neuronal activity, but rather disrupts pathological firing patterns by loosely entraining neuronal activity [77]. Similarly, research in monkeys has reported that HFS in the GPe does not lead to complete inhibition, but instead induces a complex restructuring of the temporal structure of neuronal activity [78]. This complex pattern change may be related to the therapeutic effect of DBS, but the exact mechanism remains to be elucidated and verified.

In summary, these findings suggest that the local response to DBS is influenced by both variations in synaptic inputs and alterations of intrinsic neuronal excitability through manipulation of membrane potential and ion channels to normalize firing patterns (Fig. 2). The intrinsic excitability of a neuron and synaptic efficacy, which represents the capacity of a presynaptic input to influence postsynaptic output, often work together to modify neural circuit function [27, 79, 80]. For instance, HFS can modify the temporal firing pattern of neurons in GPe and GPi, which underpins the beneficial effects of STN-DBS in PD [81]. The cortex and the direct cortical-STN projections, known as the hyperdirect pathway, are also potential components of the therapeutic mechanisms of STN-DBS [82]. In the following sections, we will discuss the antidromic and orthodromic effects of DBS through synaptic adaptations.

### Orthodromic and antidromic effects of DBS

As discussed previously, DBS can affect various neural elements, including soma, axons, and dendrites of neurons. Studies have shown that DBS activates axons and dendrites in the stimulation region, increasing the frequency of action potential (AP) output from the soma of neurons [83]. Computational models have suggested that axons and dendrites have lower stimulation thresholds than the soma [84], suggesting that stimulation primarily affects axons and dendrites in the vicinity of the electrode. Therefore, most somatic effects are likely due to the propagation of stimulation effects from their local dendritic membranes [85, 86].

A computational model [87] proposes that if the stimulus is strong enough, it triggers APs that propagate orthodromically to the distal end of the cell body facilitating neurotransmitter release, and also propagate antidromically to activate upstream neurons [88]. In addition, the AP first reaches the base of the axonal arbor and then bifurcates at various branching points, eventually invading the entire axonal arbors and reaching all terminal points (Fig. 1). This leads to neurotransmitter release not only at the stimulation site but also at other terminal sites, illustrating multiple effects of DBS [81, 87] (Table 2).

**Table 2:** Orthodromic and antidromic effects of DBS

Species	Stimulation target	Stimulus parameter	Effects in the distant regions	Citations
PD rhesus monkeys (in vivo)	STN	HFS (210 μs, 1.8 and 3 V, 136 Hz, for 5 min)	Increased mean discharge rate and stimulus-synchronized regular firing pattern in GPe and GPi neurons	[81]
PD rhesus monkeys (in vivo)	STN	HFS (136 Hz)	Inhibited VA/VLo neurons and activated VPLo neurons; reduced burst activity in VA/VLo neurons; conversed oscillatory activity in VA/VLo and VPLo neurons	[112]
PD rhesus monkeys (in vivo)	STN	HFS (125 μs, 0.2 mA 130 Hz, for 4 h; 120 μs, 2.1 V, 130 Hz, for 4 h)	Activation of M1 waned over time, but synchronization of spontaneous spiking in M1 was significantly reduced during DBS	[110]
PD and normal rats (in vivo)	STN	HFS (60 μs, 10–1000 μA, 130 Hz, for 5 s)	Decreased activity of SNr neurons and increased activity of VL neurons	[42]
PD and normal rats (in vivo)	STN	HFS (0.1 ms, 0.08–0.26 mA, 40–160 Hz)	Induced antidromic spiking of deep layer cortical neurons; triggered a dampened oscillation in cortex	[106]
PD rats (in vivo)	STN	HFS (125 Hz, for 5 min)	Increased spontaneous firing and decreased episodes of burst firing of the CxFn in the motor cortex	[88]
PD mice (in vivo)	STN	HFS (60 μs, 2–4 V, 130 Hz, for 2 min)	Normalized pathological hyperactivity of motor cortex pyramidal cells	[82]
PD mice (in vivo)	STN	HFS (60 μs, 200 μA, 60 and 100 Hz)	Increased activity of SNr and M1 neurons	[45]
Normal rats (in vivo)	STN	HFS (60 μs, 300 μA, 130 Hz, for 5 s)	Decreased activity in 91% of SNr cells and 80% of GPi cells but activated 100% of GP cells	[94]
Normal rats (ex vivo)	STN	HFS (100 μs, 130 Hz, for 30 s)	Increased spontaneous spiking in half of SNr neurons while decreased activity in the other half	[96]
Normal rats (in vivo)	STN	Electrical stimulation (69 μs, 100 μA, 0.5–130 Hz, for 300 s)	Produced some entrainment of firing in PPN	[101]
PD mice (in vivo)	STN	Optical HFS using ChR2 (100–130 Hz)	Reduced theta and alpha and increased gamma power in M1	[108]
PD patients (in vivo)	STN	Electrical stimulation (1, 2 and 3 mA, 1 Hz for 30 s or 10 Hz for 30 s)	Activated the SMG, premotor and motor regions	[100]
PD and dystonia patients (in vivo)	STN and GPi	HFS (0.5 s, 4 μA, 200 Hz)	Inhibited firing in the GPi and the SNr	[227]
PD monkey	GPi	HFS (0.2 ms, 300 μA, 120 Hz)	Decreased and increased discharge frequency in 77% and 16% of thalamic neurons, respectively; reduced bursting in thalamic neurons	[113]

*CxFn* Corticofugal projection neurons, *GP* Globus pallidus, *GPe* Globus pallidus externus, *GPi* Globus pallidus internus, *HFS* High-frequency stimulation, *M1* Primary motor cortex, *PPN* Pedunculopontine nucleus, *SMG* Superior marginal gyrus, *SNr* Substantial nigra pars reticulate, *STG* Superior temporal gyrus, *STN* Subthalamic nucleus, *VA/VLo* Ventralis anterior /ventralis lateralis pars oralis, *VL* Ventrolateral thalamus, *VPLo* Ventralis lateralis posterior pars oralis

As an intrinsic property, electrical stimulation propagates in multiple directions, thus DBS can modulate neural circuits in various disease states. The sustained changes in neural activity induced by DBS may trigger adaptive changes within the nervous system, including activity-dependent synaptic adaptations in clinical settings [31–33]. This involves the reconfiguration of neuronal and synaptic components and the homeostatic regulation of neural circuit function [27].

A substantial body of research supports this synaptic adaptation theory, with observations indicating that DBS normalizes the distribution of corticostriatal glutamatergic terminals, thereby altering striatal glutamatergic neurotransmission in animal models [89]. In addition, DBS has been shown to modulate key components of the motor cortico-striato-thalamo-cortical loop in humans [90]. The enhancement of inhibitory synaptic transmission [91] and the restoration of intracortical inhibition associated with motor improvements [92] have also been reported in DBS studies, highlighting the role of adaptation-related mechanisms in its clinical effects.

#### Orthodromic and antidromic effects of STN-DBS

**Fig. 3:**
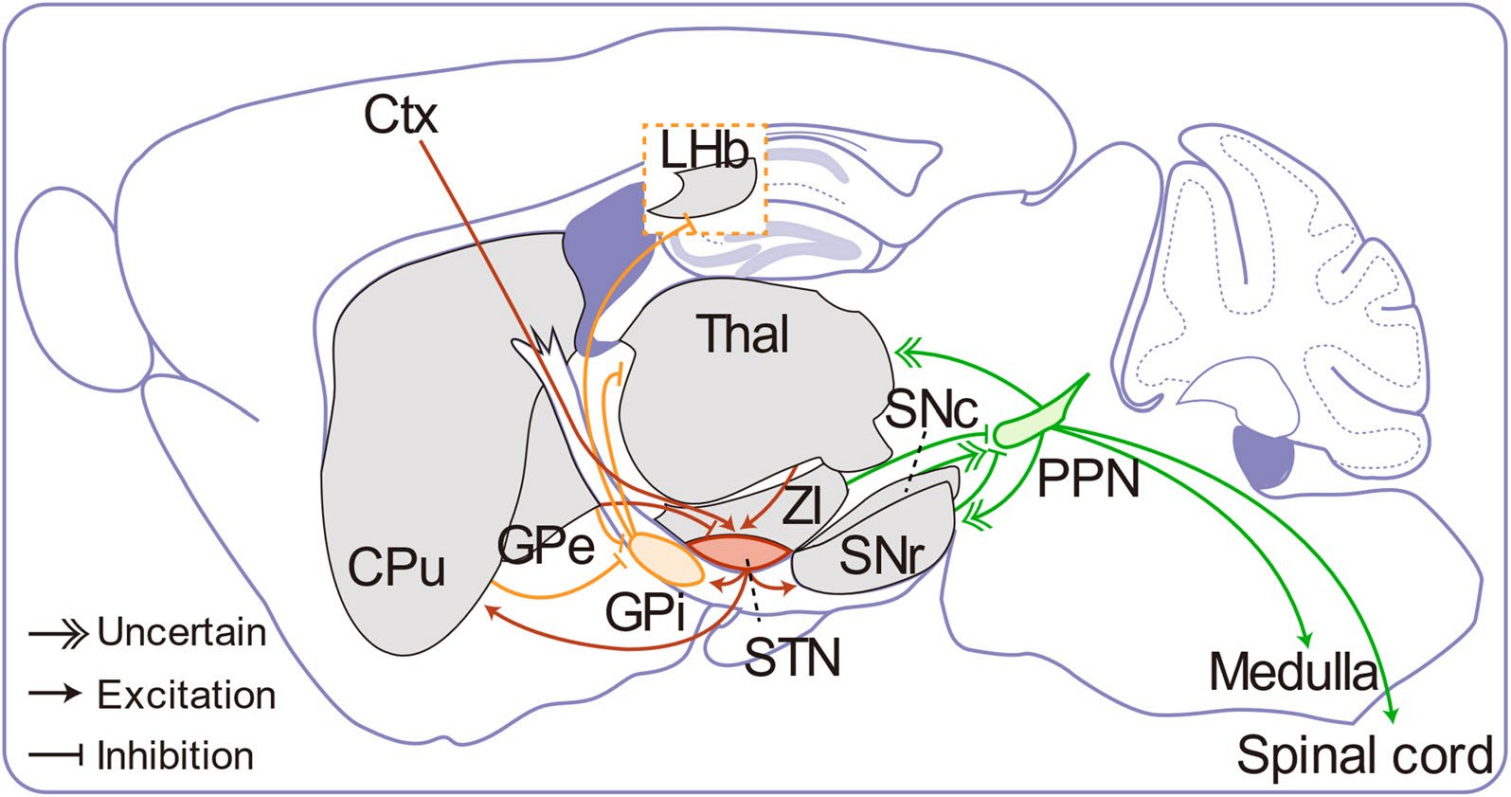
Schematic of common DBS targets and their connections in mice. The subthalamic nucleus (STN) and the globus pallidus internus (GPi) are the primary DBS targets in PD [5–8]. The STN receives excitatory input from the cortex, referred to as the hyperdirect pathway [35], and from thalamic areas like the parafascicular nucleus [120]. Its main inhibitory input comes from the globus pallidus externus (GPe), contributing to the indirect pathway. STN neurons are primarily glutamatergic and project efferent fibers to the GPi and substantia nigra pars reticulata (SNr) to convey motor information. Notably, STN neurons also project to the caudate putamen (CPu), as demonstrated by viral tracing experiments in mice [225]. The GPi primarily receives glutamatergic afferents from STN and GABAergic input from the GPe and CPu. GPi sends GABAergic efferents to the thalamus and lateral habenula (LHb) [130]. The pedunculopontine nucleus (PPN) is a component of the mesencephalic locomotor region and is targeted to address gait and postural instability issues [12, 13]. In addition to its descending projections to the medulla and spinal cord, PPN neurons project to multiple ascending targets, including the thalamus and several basal ganglia components. These projections comprise a mixture of cholinergic and noncholinergic afferents [226]. A significant portion of inputs to the PPN originates in brainstem and midbrain structures, including the substantia nigra pars compacta (SNc) and SNr. PPN neurons also receive direct input from the zona incerta (ZI) in the hypothalamus [163]

The STN controls two basal ganglia output nuclei: the GPi and the substantia nigra pars reticulata (SNr) [35]. This suggests that the effects of STN-DBS are related to the regulation of STN neurons projecting to the GPi and the SNr (Fig. 3). Electrophysiological data have illustrated the differences between STN-SNr and STN-GPi neurons in terms of their synaptic inputs, responses to electrical stimulation, and adaptations under PD conditions [93]. A prevalence of inhibitory synaptic inputs is more evident in STN-GPi neurons than in STN-SNr neurons. In PD mice, 6-hydroxydopamine (6-OHDA) lesioning disrupted the inhibitory inputs to STN-GPi neurons. This alteration reversed the predominance of inhibitory over excitatory inputs in STN-GPi neurons but did not affect synaptic inputs in STN-SNr neurons. Prolonged electrical stimulation enhanced inhibition and reduced excitation in both STN-SNr and STN-GPi neurons [93]. Consistent with this, in vivo recordings confirmed that STN-DBS led to the inhibition of neurons in GPi and SNr [42, 94], consequently suppressing the basal ganglia output and relieving the ventrolateral motor thalamic nucleus activity, thereby ameliorating PD symptoms [42]. However, in an experiment with two Parkinsonian rhesus monkeys, subthalamic stimulation elicited short-latency excitatory responses that caused a tonic increase in the average firing rate in the GPi and the GPe [81]. Furthermore, GCaMP (genetically encoded calcium indicator) fiber photometry in PD mice showed increased SNr activity during STN-DBS [45]. Although different experimental conditions may lead to different conclusions, this evidence supports that STN-DBS likely acts by disrupting neuronal activity patterns within the STN rather than by direct inhibition or antidromic activation [95]. Notably, similar to the complex response of STN neurons during HFS, spontaneous spiking of neurons in the SNr also exhibits variability [96].

As downstream nuclei of the STN (Fig. 3), the SNr, GPi, and ventral pallidum (VP) play a role in mediating PD-related pain, a prevalent and distressing non-motor symptom affecting 30%–95% of patients [97]. In normal mice, unilateral optogenetic activation (channelrhodopsin-2, ChR2) of the STN-SNr projections reduces the thermal but not the mechanical pain threshold, whereas stimulation of the STN-GPi and STN-VP projections reduces the mechanical but not the thermal pain threshold [98]. In PD models, optogenetic inhibition (NpHR) of the STN-SNr projection attenuates mechanical and thermal pain hypersensitivity, whereas photoinhibition of STN-GPi and STN-VP projections reduces only mechanical but not thermal pain hypersensitivity. These results suggest that subpopulations of the STN projection neurons control the perception of different pain stimuli. While STN-DBS may partially alleviate pain by improving motor function in PD, it is also essential to modulate individual STN projections linked to the nociceptive network to achieve its analgesic effects.

In addition to its orthodromic effects, several studies have suggested that DBS can also modulate neuronal activity in upstream nuclei through antidromic stimulation (Fig. 3) [82, 99–101]. While synaptic inputs play a minor role in maintaining spontaneous firing in STN neurons [102, 103], they play a critical role in shaping the firing rate and pattern [104, 105]. Notably, afferent axons projecting to STN neurons, particularly those within the hyperdirect pathway, are directly stimulated and highly implicated in the therapeutic effects of STN-DBS [106, 107].

Studies have shown that the layer-5 primary motor cortex (M1) neurons project extensively to the STN [108, 109]. Optical HFS (ChR2-E123T/T159C) of these projections significantly ameliorates hypokinesia and bradykinesia [108]. The spontaneous firing rate of M1 pyramidal cells increases in rats lesioned with 6-OHDA [82]. Interestingly, DBS can normalize the pathophysiological hyperactivity of motor cortex pyramidal neurons while inhibiting PV^+^ and activating somatostatin (SST)-expressing (SST^+^) GABAergic interneurons [82]. This study further demonstrated that direct optogenetic activation (ChR2-H134R) of cortical SST^+^ interneurons can mimic the effects of DBS and alleviate motor symptoms in a PD mouse model, suggesting that STN-DBS may recruit cortical GABAergic networks to inhibit the hyperactivity of cortical-subthalamic projections.

Paradoxically, another study in rats found that the average spontaneous firing rate of corticofugal projection neurons (CxFn) was reduced after 6-OHDA treatment, accompanied by increased episodes of burst firing. STN-DBS at 125 Hz significantly increased the spontaneous firing of CxFn, disrupting the dominance of the beta rhythm [88]. Antidromic activation of M1 during STN-DBS has been reported to contribute to the disruption of synchronization in cortical neuronal populations in Parkinsonian non-human primates [110]. However, the antidromic activation diminished over time and was not observed during GPi-DBS, which had similar therapeutic effects as STN-DBS, raising doubts about the mechanisms underlying the therapeutic effect of DBS. These inconsistencies in conclusion may be due to differences in experimental animals, models, stimuli, and measurement methods. Nevertheless, these studies suggest that STN-DBS, to some extent, regulates cortical neuronal activity through antidromic transmission.

Within the STN, the hyperdirect and indirect pathways serve as the main motor inhibitory circuits in the basal ganglia [35]. The hyperdirect pathway predominantly conveys glutamatergic inputs from the motor cortex to the STN, while the indirect pathway primarily transmits GABAergic inputs to the STN from the GPe. An intrinsic homeostatic mechanism in the STN has been identified to balance cortical excitation by adjusting the strength of GPe inhibition [111]. Stimulation of the motor cortex-STN inputs by optogenetic activation (ChR2-H134R) of motor cortical projection neurons induces heterosynaptic long-term potentiation (LTP) of GPe-STN transmission through NMDARs. This process may promote pathological activity after dopamine depletion [111]. In conclusion, DBS normalizes pathological hyperactivity of the motor cortex and indirectly inhibits GPe-STN transmission through heterosynaptic regulation of the hyperdirect and indirect pathways. NMDAR-dependent processes in neurons receiving afferents from the STN are likely a cellular mechanism by which STN-DBS exerts its therapeutic effects.

The thalamus is a critical node in the basal ganglia network, and several studies indicate that STN-DBS can affect the firing rate and the bursting activity of thalamic neurons [112, 113]. Among the different thalamic nuclei, the parafascicular nucleus (Pf) has been identified as a critical player in modulating basal ganglia activity and mediating the therapeutic effects of STN-DBS [114, 115]. The Pf is involved in the regulation of striatal function and plays a critical role in learning, arousal, and behavioral flexibility [116, 117]. Pf neurons project to both the STN and striatum, suggesting that the Pf-STN pathway may contribute to the clinical benefits of STN-DBS [118, 119].

A study shows that optogenetic stimulation (ChR2-H134R) of Pf projections to the STN leads to improved motor function, whereas stimulation of the Pf-striatum cell body or terminal does not have the same effect [119]. Unilateral or bilateral optogenetic stimulation of the Pf-STN terminal significantly improves locomotion and alleviates severe akinesia in a bilateral 6-OHDA PD model [119]. In addition, optogenetic enhancement (oChIEF) of the Pf-STN circuit using the optical LTP approach restores motor learning. Notably, inhibition of PV^+^ STN neurons prevents this LTP-based recovery, highlighting the critical role of PV^+^ STN neurons in this rescue process [120]. These findings suggest that the Pf-STN pathway provides a circuit mechanism that may elucidate the clinical efficacy of STN-DBS in alleviating motor symptoms of PD.

#### Orthodromic and antidromic effects of GPi-DBS

GPi, one of the commonly targeted brain regions for DBS, has shown therapeutic effects like STN. Studies have demonstrated that GPi-DBS can reduce the activity of neurons within the STN. The suppression could be attributed to the activation of fibers that originate in the GPe and pass through the GPi. Recently, researchers have discovered a phenomenon called evoked resonant neural activity (ERNA) occurring in GPi-DBS [121, 122]. ERNA is a HFS-evoked response typically occurring at 200 to 500 Hz, and is associated with synchronized patterned neuronal inhibition. Additionally, ERNA has also been reported in STN-DBS [123–128], and a biophysical model suggests that it results from the reciprocal connections between the STN and GPe [125]. GPi-DBS has the potential to activate fibers within the STN-GPe loop or affect axon collaterals [122], leading to the possible indirect triggering of ERNA. It seems that GPi-DBS is effective in influencing the activity of STN but an alternative theory states that both STN-DBS and GPi-DBS produce comparable modulatory effects on an "overlapping" functional network in PD patients [122, 129]. This hypothesis is supported by practical research that discovered remarkably similar connectivity profiles associated with STN-DBS and GPi-DBS [129].

In practice, while both GPi and STN are recommended as potential DBS targets, their clinical outcomes differ. STN-DBS typically results in a greater reduction of levodopa usage, whereas GPi-DBS is linked with a lower frequency of neuropsychiatric side effects [15]. A viral genetic tracing study in mice showed that neurons in the entopeduncular nucleus (EP, analogous to GPi in human) gather inputs from both the striatum and GPe. They then relay the inputs prominently to the lateral habenula (LHb) and the ventro-anterior lateral thalamus/ventro-medial thalamus (VAL/VM) [130] (Fig. 3). The neurons situated in the EP provide inhibitory input to the VAL/VM thalamus to control movement. Conversely, when they are inhibited by upstream basal ganglia nuclei, the movement is allowed [131]. Electrophysiological studies in primates and humans with PD suggest that increasing the firing rates of GPi neurons could lead to the development of motor deficits associated with the condition, likely due to VAL/VM thalamus inhibition and decreased basal ganglia output [40, 132, 133]. LHb neurons are arranged to receive EP input and project to the rostral medial tegmental area, which innervates the ventral tegmental area and is involved in aversive conditioning [134]. Consistent with this study, electrophysiological studies in primates have demonstrated that GPi neurons, projecting to the LHb, respond to reward-related signals and some sensory stimuli [135].

Therefore, with regards to its orthodromic effects, GPi-DBS has the potential to regulate neuron activity in the VAL/VM thalamus, thus improving motor function. Additionally, it may also yield emotional and neuropsychological benefits by impacting the LHb activity.

### Position-dependent therapeutic effects

**Fig. 4:**
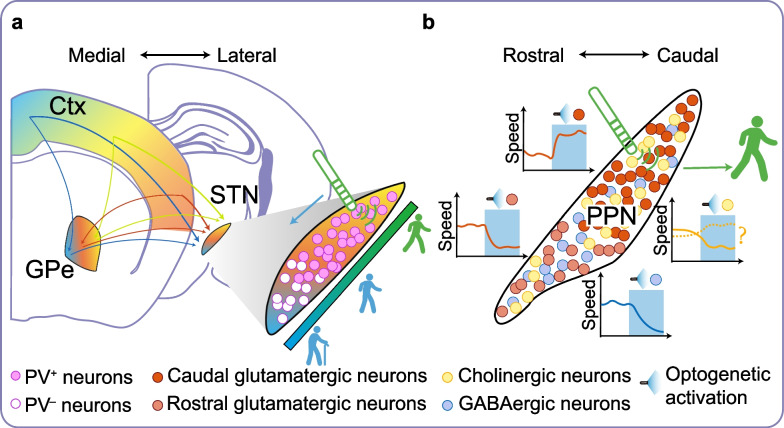
Position-dependent therapeutic effects of DBS for PD. **a** Topologically determined position-dependent effects (exemplified by STN-DBS). In mice, the STN receives inputs from both the cortex and the GPe. These inputs exhibit a topographically graded organization, forming the hyperdirect and indirect pathways, respectively [150]. Furthermore, a topographical organization exists between the cortex and GPe. To elaborate, the posterolateral to anteromedial regions of the STN receive projections from various cortical areas, including sensorimotor, association, and limbic regions [136, 147, 148]. Within the STN, there is a distinct distribution of PV^+^ glutamatergic neurons, primarily clustered in the dorsolateral and middle regions. These neurons exhibit unique burst firing patterns and may contribute to excessive burst firing observed in PD [150]. Consequently, clinical benefits are typically observed when DBS electrodes are precisely positioned within the dorsolateral sensorimotor area [136–139]. **b** Neuronal population-determined position-dependent effects (exemplified by the PPN-DBS). The PPN is an integral component of the mesencephalic locomotor region, characterized by the spatial distribution of glutamatergic, GABAergic, and cholinergic neurons [159, 160]. Among these, glutamatergic neurons represent the major subpopulation. Activation of caudal glutamatergic neurons promotes locomotion [155, 162, 163], while their rostral counterparts induce locomotor arrest [162]. GABAergic neurons are slightly more concentrated in the rostral PPN [161] and tend to decrease the locomotor speed when activated [155, 163]. Cholinergic neurons outnumber GABAergic neurons, yet their influence on locomotion is less clear, with reported effects spanning from improvement to suppression of movement upon optogenetic activation [155, 163]. The specific distribution of these neuronal subpopulations likely underlies the rationale for targeting the caudal PPN as the optimal stimulation site [142–145]

Due to the position-dependent nature of DBS therapy, the positioning of electrodes and their active contacts is a crucial parameter in DBS treatment, which requires precise programming by physicians. Clinically, the electrodes are often placed in regions that yield the maximal DBS benefits, such as the dorsolateral STN [136–139], the posterolateral GPi [140, 141], and the caudal PPN [142–145]. These regions have distinct features of circuit connectivity and cellular composition, which should be seriously considered when tailoring electrode placement (Fig. 4).

#### Topological factor for position-dependent effects

The position-dependent effects are influenced by topological factors (Fig. 4a). Despite the STN and GPi being relatively small nuclei in primates and rodents, several human studies have revealed the presence of three territories within these regions: sensorimotor, associative, and limbic territories [136, 146, 147]. For instance, in the case of the STN, anatomical-functional subregions extending from the posterolateral to the anteromedial parts of the nucleus receive projections from sensorimotor, association, and limbic areas in the cortex [136, 147]. This organization aligns with findings from anterograde tracing studies in primates [148]. Single-cell recordings from PD patients have also identified neurons with sensorimotor responses in the dorsolateral region of the STN [149]. This topographical organization of the STN supports the structural basis for information processing from the cortex to the basal ganglia. Recent mouse studies have further confirmed this organization, revealing a graded distribution of cortical projections to the STN with a notable degree of overlap along its longitudinal axis [150], in line with earlier studies [148, 151]. The convergent projection patterns within the STN reinforce the clinical efficacy of DBS in the dorsolateral STN, which is associated with sensorimotor functions.

Similarly, the GPi also exhibits a topological distribution [146]. The anterior region of GPi is associated with limbic territories and associative connectivity, while the posterolateral regions of the nucleus are linked to sensorimotor functions [140, 152]. Individualized treatment planning, focusing on identifying the sensorimotor regions of the GPi, particularly its posterolateral aspect, has been demonstrated to enhance the alleviation of PD motor symptoms through DBS [140, 153]. This underscores the importance of the topological structure when determining electrode implantation sites in DBS therapy.

#### Neuronal population factor for position-dependent effects

The position-dependent effects of DBS are influenced not only by topological factor but also by the composition of neuronal populations within targeted regions (Fig. 4b). A close examination of the STN revealed heterogeneity in neuronal population, although it is generally considered a homogeneous glutamatergic nucleus. Serial multiplex single-molecule fluorescence in situ hybridization data showed the presence of two populations: PV^+^ and PV^−^ neurons in the STN. The glutamatergic PV^+^ neurons predominantly occupy the dorsolateral and middle portions of the STN and exhibit characteristics of phasic burst firing compared to the PV^−^ subpopulation [150]. Electrophysiological recordings in PD patients have further corroborated this finding, showing spatial distribution of burst activity mainly in the dorsal region of the STN [154]. This burst firing, along with beta oscillations, is a hallmark of PD. STN-DBS mitigates these pathophysiological patterns that are associated with motor symptoms in PD [69], suggesting that the glutamatergic PV^+^ neurons are the source of excessive burst firing in PD. Their distribution in the dorsolateral and middle parts of STN likely underlies the position-dependent effects observed in STN-DBS [12, 13].

Similarly, for PPN-DBS, the position-dependent effects are pivotal for addressing freezing gait and postural abnormalities in PD patients who are resistant to dopaminergic treatments [155–158]. However, clinical outcomes of PPN-DBS can be variable [158], possibly due to the nonspecific electrical stimulation of different PPN populations and regions. The PPN consists of spatially distributed glutamatergic, GABAergic, and cholinergic neurons [159, 160], with the glutamatergic neurons as the major neuronal subpopulation. These neurons are functionally diverse and produce different motor responses upon activation [161]. For instance, the caudal vesicular glutamate transporter 2 (VGLUT2)-expressing (VGLUT2^+^) neurons promote locomotion within an exploratory speed range [155, 162, 163], while those in the rostral PPN induce locomotor arrest. The GABAergic neurons are more concentrated in the rostral PPN [161] and decrease locomotor speed when optogenetically activated (ChR2) in rodents [155, 163]. The cholinergic neurons, while more abundant than GABAergic neurons, exhibit uncertain effects on locomotion, as both improvement and suppression of movement upon optogenetic activation have been reported in mice [155, 163].

Studies indicate that the motor-enhancing effect of PPN-DBS is specifically attributed to the caudal PPN [142–145]. Chemical genetic activation (hM3Dq) of the caudal VGLUT2^+^ PPN neurons can rescue movement deficits in PD mice [160], and DBS in the caudal PPN improves gait parameters in PD rats [142]. This is consistent with clinical findings that DBS in the caudal PPN enhances gait freezing and postural stability in PD patients [144]. Importantly, the glutamatergic PPN neurons projecting to different targets, such as the SNr or spinal cord, may underlie various DBS effects, influencing forelimb movements, behavior, or body extension, depending on the specific projection [156]. As a result, the therapeutic effects of PPN-DBS on freezing gait and postural balance may depend on the specific subsets of PPN neurons being stimulated.

In summary, both the topological structure and the composition of neuronal subpopulations contribute to the spatial distribution of inputs and outputs in targeted brain regions. These factors collectively determine the position-dependent therapeutic effects of DBS.

### DBS programming-dependent therapeutic effects

DBS programming, the adjustment of electrical stimulation parameters to optimize clinical benefit for individual patients, is a critical aspect of DBS. Physicians must carefully fine-tune parameters such as frequency, pulse width, voltage, and electrode contact to achieve the best symptom relief with minimal side effects [164]. The selection of the active contact is predominantly influenced by position-dependent effects, and consequently, it affects communication within the pertinent neural circuits. The other programming parameters also play a vital role in modulating neural circuitry and synaptic plasticity.

#### Frequency-dependent therapeutic effects

Frequency is a crucial parameter in DBS programming. Studies have found that the magnitude of the beneficial effect in PD patients is most pronounced within the frequency range of 130–185 Hz, with a progressive improvement in motor symptoms as the frequency increases from 50 to 130 Hz [165]. Similar observations have been made in PD mice undergoing STN-DBS, showing that the movement speed scales linearly with frequency up to approximately 120 Hz. This phenomenon mirrors the response seen in PD patients undergoing STN-DBS. The rationale behind these observations lies in the synaptic depression caused by repetitive higher frequency stimulation, which weakens synaptic transmission strength and suppresses somatic firing in postsynaptic neurons. The application of halorhodopsin (NpHR) as an optogenetic tool for inhibiting postsynaptic neurons in the GPi or STN has shown remarkable promise in improving motor symptoms in hemiparkinsonian animal models [166–169]. These effects are consistent with the therapeutic benefits observed with HFS-DBS in PD patients.

However, when attempting to directly activate local excitatory STN neurons using optogenetic methods like ChR2, results were less favorable, with only minimal changes in rotational behavior and even motor deficits in the contralateral limb [107, 170]. These findings suggest that optogenetic stimulation, particularly with ChR2, cannot precisely replicate the effects of STN-DBS. This limitation is attributed to the relatively slow opening and closing kinetics of ChR2, which cannot generate firing rates > 100 Hz in the STN or drive glutamate release at rates greater than 100 Hz [171, 172]. To potentially bridge this gap, faster optogenetic actuators like fast channelrhodopsin (i.e., ChR2-E123T/T159C) or Chronos have been proposed [108, 173]. Optogenetic STN stimulation using Chronos at a frequency of 130 pulses per second demonstrated a reduction in pathological circling behavior and an improvement in forelimb stepping deficits, mirroring the effects of electrical DBS [173]. Faster optogenetic actuators have the potential to generate higher overall firing rates and greater firing rate fidelity than ChR2. Consequently, the therapeutic effects of DBS may be more closely tied to the stimulation frequency when using these faster actuators [171, 174]. Furthermore, a study by Yu et al. [173] noted significant differences in the absolute changes in the firing rates of responsive STN neurons across several stimulation frequencies using optogenetic stimulation with Chronos. In an optogenetic experiment, the targeted cells and their axons, rather than afferent or passing axonal fibers, were selectively activated [175]. This implies that different stimulation frequencies have varying effects on the postsynaptic neuronal intrinsic excitability.

Recent human studies have proposed that the shape and the amplitude of ERNA, generated by DBS, also depend on the frequency and duration of stimulation [124, 125]. The steady states of ERNA frequency and amplitude do not immediately return to baseline when STN-DBS is turned off, and it takes several seconds for these parameters to normalize [124]. Higher stimulation frequencies have been associated with significantly longer silent periods after stimulation [44]. These slow temporal dynamics in the recovery period may be linked to the time needed to replenish presynaptic vesicle pools, which affect the synaptic transmission fidelity [58]. In essence, the gradual changes observed in ERNA may be correlated with the progressive deterioration of synaptic transmission fidelity [124, 176]. While neurons and the axons of afferent and efferent pathways can potentially keep pace with HFS for an extended period, synaptic resources are more likely to be depleted within seconds to minutes. This depletion results in a functional disconnection between the STN and the broader basal ganglia network [124].

Another hypothesis regarding synaptic depression suggests the involvement of presynaptic metabotropic GABA_B_ receptors, which lead to longer-lasting inhibitory effects at higher frequencies compared to lower frequencies [44]. This mechanism relies heavily on the regulation of Ca^2+^ conductance. Reductions in Ca^2+^ conductance, both on autoreceptors located on GABA-releasing terminals and on heteroreceptors in neighboring terminals, are thought to be induced by HFS. This ultimately results in the inhibition of neurotransmitter release [59, 177–179]. However, it is important to note that human studies have yet to definitively elucidate these adaptation mechanisms at the molecular and synaptic levels [31–33]. Consequently, these explanations remain speculative and need further validation through additional research involving animal models.

#### Pulse width/intensity-dependent therapeutic effects

The therapeutic efficacy of DBS is profoundly influenced by the spatial distribution of the stimulation field in relation to the brain's anatomy [180–182]. Clinical studies have lent support to this idea, indicating that the volume of tissue activated, a parameter modifiable through DBS settings, including pulse width and voltage or current intensity titration, can be instrumental in altering the range of stimulated nervous tissue, consequently impacting the clinical outcomes of DBS [137, 183, 184].

Preclinical data reinforce these findings. The effectiveness of STN-DBS, as measured by improvements in movement speed in PD mice, has exhibited a linear correlation with pulse width and current intensity [139]. Nevertheless, once pulse width values reach a certain threshold, lower stimulus intensities may be needed to achieve the desired clinical effect [185]. It is worth noting that dyskinesia can emerge as a side effect of HFS in humans [186], and similar effects have been demonstrated in mice that HFS, alongside increased pulse width and current, can lead to severe dyskinesia in vivo [187].

In vitro electrophysiological studies have illuminated the role of pulse width in determining the type of neuronal response elicited by HFS in the GPi. Low charge density HFS (60 μs) primarily induces excitation, while high charge density HFS (400 μs) triggers a distinct subtype of excitation characterized by late inhibition, which involves glutamatergic and cholinergic modulation, as well as Ca^2+^-activated non-specific cation channels [46]. Furthermore, elevating the intensity of HFS has been found to extend the duration of excitation in the excitation-only after-effect [46]. The amplitudes of ERNA and the silent periods observed during HFS-DBS also display a positive relationship with pulse width and intensity [124, 188]. These observations underscore the pivotal role of programming parameters in modulating synaptic adaptions.

#### Long-lasting effects mediated by optimized programming

While DBS has demonstrated symptomatic efficacy in PD, its effects are transient and vanish once the stimulation is discontinued, leading to a swift return of motor symptoms [139, 189]. However, recent investigations have shown that optimized programming, facilitated by patterned electrical stimulation, can yield enduring therapeutic benefits. Coordinated reset DBS (CR-DBS), an innovative DBS approach, is under investigation in preclinical and clinical studies [190] and have shown potential to induce sustained therapeutic improvements in Parkinsonian symptoms, even after stimulation cessation [191–194]. CR-DBS aims to reconfigure the neuronal connectivity therapeutically by modulating synaptic plasticity, particularly spike timing-dependent plasticity (STDP) [195–197]. This approach reduces the coincidence rates, resulting in a decrease of synaptic weights due to STDP, making the network unlearn pathological connectivity and synchronicity [198].

Furthermore, Spix and colleagues have delineated a precise DBS stimulation protocol with long-term efficacy in mice, highlighting distinct responses of two types of neurons in the GPe to electrical stimulation [199]. The GPe, a basal ganglia nucleus, maintains connections with various brain regions, including the thalamus, amygdala, brainstem, and cortex [200, 201], and plays a role in abnormal neural dynamics seen in PD [202]. Mastro et al. [201] have demonstrated that optogenetic activation of PV^+^ neurons using ChR2 and inhibition of Lim homeobox protein 6 (Lhx6)-expressing (Lhx6^+^) neurons via Arch (a light-activated inhibitory proton pump), two subpopulations in the GPe with distinctive intrinsic physiological and projection properties, ameliorate locomotor deficits in dopamine-depleted mice four hours following stimulation [203]. Building on this, Spix et al. employed a specific electrical stimulation mode (175 Hz, 200 ms) utilizing brief bursts to effectively segregate the responses of PV^+^ and Lhx6^+^ GPe neurons. Burst stimulation not only improves bradykinesia in 6-OHDA-lesioned mice but also provides long-lasting therapeutic benefits that persist for hours post-stimulation [199]. These findings suggest that the induction of sustained behavioral improvement arises from frequency-dependent, cell-type-specific activation or inhibition, specifically, an increase in the firing rate of PV^+^ GPe neurons relative to Lhx6^+^ GPe neurons.

**Fig. 5:**
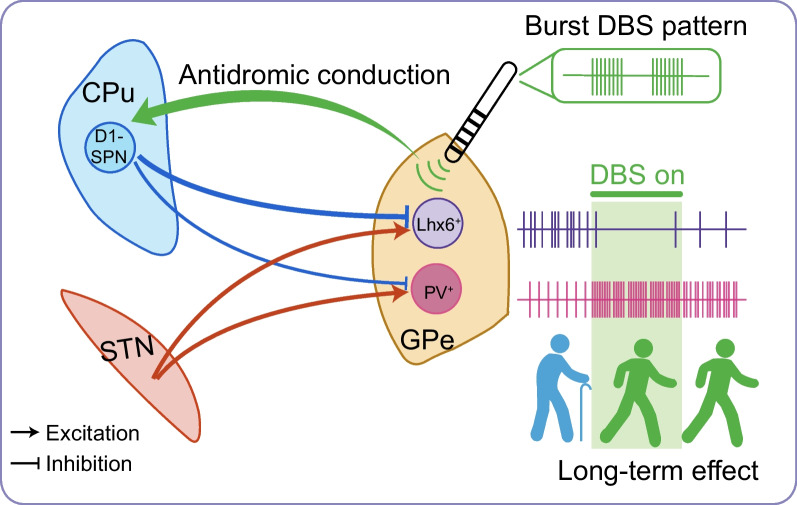
Optimized programming of DBS produces long-lasting effects. An example of GPe-DBS with population-specific neuromodulation that prolongs therapeutic benefits [199]. Both PV^+^ GPe and Lhx6^+^ GPe neurons receive excitatory inputs from STN to a similar degree. However, a distinction arises in their inhibition patterns originating from D1-SPN afferents. Lhx6^+^ GPe neurons experience proportionally greater inhibition from these afferents compared to PV^+^ GPe neurons. A highly precise electrical stimulation mode (175 Hz, 200 ms) with brief bursts is designed to bias towards antidromic activation of D1-SPNs, resulting in more potent inhibition of Lhx6^+^ GPe, while simultaneously exciting PV^+^ GPe neurons. Consequently, the firing rates of PV^+^ GPe neurons exceed those of Lhx6^+^ GPe neurons, which plays a crucial role in ameliorating bradykinesia in 6-OHDA-lesioned PD mice. Notably, these improvements persist long after stimulation. While the precise mechanism responsible for the extended therapeutic effects achieved through GPe-DBS with relative cell-specificity remains elusive, it is conceivable that this specific stimulation pattern bears similarities to certain forms of DBS, notably adaptive and coordinated reset DBS, both of which have shown the ability to produce enduring therapeutic benefits [191–194]

The differing circuit properties of PV^+^ and Lhx6^+^ GPe neurons are one mechanism underpinning their distinct firing responses and the sustained effects observed. Despite receiving similar levels of excitatory input from the STN, Lhx6^+^ GPe neurons receive proportionally more inhibition than PV^+^ GPe neurons from the D1-dopamine receptor-expressing spiny-projection neuron (D1-SPN) afferents. The specific electrical stimulation applied is likely skewed toward the antidromic activation of D1-SPNs, thereby producing more potent inhibition of Lhx6^+^ GPe neurons than PV^+^ GPe neurons. This disruption in the network's balance through the stimulation of distinct neuronal subpopulations results in sustained therapeutic benefits (Fig. 5) [199]. These findings may serve as a basis for understanding the cell-type-specific mechanisms of DBS and exploring tailored stimulation strategies for potential clinical applications.

### Optimization of DBS treatment

In recent decades, DBS is emerging as a pivotal treatment for various refractory movement and psychiatric disorders. While DBS has demonstrated efficacy and safety, it also has challenges including diminishing efficacy over time and the occurrence of adverse effects [187, 204]. To address these issues and enhance therapeutic outcomes, researchers have been exploring novel stimulation methods, including innovative waveform shapes and patterns. However, further evaluation and refinement are necessary to fully optimize DBS treatments [205].

#### Adaptive stimulation

Adaptive DBS (aDBS) is a promising avenue for DBS application, which seeks to enhance the effectiveness and safety of DBS by dynamically adjusting stimulation parameters based on real-time feedback signals. Unlike conventional open-loop DBS, aDBS operates as a closed-loop system, which is capable of bidirectional communication and automatic parameter adjustment [206, 207]. This feature makes aDBS a potential strategy to control the symptoms of PD [208, 209] and mitigate the levodopa-induced dyskinesia [210].

To effectively implement aDBS, it is essential to identify an electrophysiological biomarker that can accurately reflect the clinical characteristics of the disease and serve as a feedback for the system. The LFP, particularly beta oscillation across the motor network, has been widely employed as a biomarker for aDBS [209, 211, 212]. However, since beta oscillation is more closely associated with rigidity and bradykinesia than with tremor [213–216], there is a pressing need for the identification of biomarkers that can capture different PD symptoms to facilitate the development of effective aDBS algorithms.

One promising candidate is the narrowband gamma activity (60–90 Hz) observed in the motor cortex and STN during dyskinesia [217]. This gamma activity shows potential as a biomarker for aDBS [218], as it is less influenced by voluntary movements and exhibits sensitivity to stimulation-induced dyskinesia, displaying a higher signal amplitude and a more favorable signal-to-noise ratio compared to beta activity [217]. Furthermore, burst firing in the STN by individual neurons has been directly implicated in PD pathophysiology and the manifestation of PD symptoms [69]. Its role in aDBS strategies warrants further exploration [219].

In summary, further refinement of biomarkers is needed to advance aDBS application. Future research endeavors may explore new biomarkers to unlock even better therapeutic outcomes through aDBS treatments.

#### Directional stimulation

Directional stimulation technology represents a promising frontier in the evolution of DBS therapy. It introduces a level of precision previously unseen in DBS treatments, achieved by manipulating or configuring electrodes with radially segmented contacts, anodes, and cathodes to guide the flow of current in specific directions. This innovative approach offers a potential for a more nuanced and adaptable stimulation field [205, 220], capable of preventing unnecessary spread or "leakage" of stimulation, thereby expanding the therapeutic window in practical DBS applications. Adverse effects of DBS often stem from its non-selective stimulation that affects nearby neurons, including surrounding structures involved in various circuit connections with diverse physiological functions.

Clinical investigations have demonstrated that directional electrodes can deliver more efficient stimulation at a given amplitude compared to omnidirectional electrodes [221–223]. The directional electrodes hold promise for enhancing DBS effectiveness while minimizing adverse effects [220, 223, 224]. Improvement of the understanding of brain anatomy and circuit projections will guide the precise targeting of DBS stimulation. Therefore, directional stimulation stands out as a pivotal direction for the development of DBS therapy.

#### More precise stimulation, more effective treatment

Recent advances in neuroscience and brain function research have provided deeper insights into the topological connections between different brain regions and the distribution patterns of distinct neuronal subgroups within various nuclei. These advances have partially illuminated the electrophysiological and circuit mechanisms underpinning the clinical effects of DBS, while also directing the development of DBS technology. The intricate anatomical complexity and circuitry interconnections among numerous brain nuclei suggest that non-selective DBS stimulation may lead to unintended clinical side effects.

The emerging cutting-edge technologies such as aDBS and directional stimulation improve DBS therapies toward delivering more efficient and targeted interventions. It is increasingly evident that leveraging more specific DBS stimulations to achieve precise modulation of neural function is a future direction of development in this field.

## Conclusions

DBS stands as a valuable treatment modality for advanced PD. In this review, we have delved into recent opto-DBS studies, shedding light on the potential mechanisms of neuronal and synaptic adaptations that underlie the efficacy of DBS in PD. Response of local neural circuits to DBS can be affected by a complicated interplay of many factors, including the distribution of presynaptic inputs, frequency-dependent synaptic depression, and the intrinsic excitability of postsynaptic neurons, which involves membrane potential dynamics and ion channel functionality. These factors collectively enable both antidromic and orthodromic modulation of neural circuits, laying the foundation for understanding the position- and programming-dependent therapeutic effects and side effects associated with DBS.

## Data Availability

The datasets used and/or analyzed during the current study are available from the corresponding author on reasonable request.

## Abbreviations

6-OHDA: 6-Hydroxydopamine
aDBS: Adaptive DBS
ChR2: Channelrhodopsin-2
CR-DBS: Coordinated reset DBS
CxFn: Corticofugal projection neurons
D1-SPN: D1-dopamine receptor-expressing spiny projection neuron
DBS: Deep brain stimulation
E/I: Excitatory/inhibitory
EP: Entopeduncular nucleus
ERNA: Evoked resonant neural activity
GABA: γ-Aminobutyric acid
GPe: Globus pallidus externus
GPi: Globus pallidus internus
HFS: High-frequency stimulation
LFP: Local field potential
LHb: Lateral habenula
Lhx6: Lim homeobox protein 6
LTP: Long-term potentiation;
NMDAR: *N*-methyl-*D*-aspartate ionotropic glutamate receptor
NpHR: Halorhodopsin
PD: Parkinson's disease
Pf: Parafascicular nucleus
PPN: Pedunculopontine nucleus
PV: Parvalbumin
SNr: Substantia nigra pars reticulata
SPN: Spiny projection neuron
SST: Somatostatin
STN: Subthalamic nucleus
VAL: Ventro-anterior lateral thalamus
VM: Ventro-medial thalamus
VP: Ventral pallidum
VGLUT2: Vesicular glutamate transporter 2
